# Molecular chaperone GRP78 enhances aggresome delivery to autophagosomes to promote drug resistance in multiple myeloma

**DOI:** 10.18632/oncotarget.3075

**Published:** 2014-12-26

**Authors:** Mohamed A.Y. Abdel Malek, Sajjeev Jagannathan, Ehsan Malek, Douaa M. Sayed, Sahar A. Elgammal, Hanan G. Abd El-Azeem, Nabila M. Thabet, James J. Driscoll

**Affiliations:** ^1^ The Vontz Center for Molecular Studies, University of Cincinnati College of Medicine, Cincinnati, OH; ^2^ Division of Hematology and Oncology, University of Cincinnati College of Medicine, Cincinnati, OH; ^3^ Department of Clinical Pathology, Faculty of Medicine, Assiut University, Assiut, Egypt; ^4^ Department of Clinical Pathology, South Egypt Cancer Institute, Assiut University, Assiut, Egypt; ^5^ Department of Cancer Biology, University of Cincinnati College of Medicine, Cincinnati, OH; ^6^ University of Cincinnati Cancer Institute, Cincinnati, OH

**Keywords:** GRP78, Aggresome+Autophagosome Pathway, Proteasome, Myeloma, Chemoresistance

## Abstract

Despite the clinical benefit of the proteasome inhibitor bortezomib, multiple myeloma (MM) patients invariably relapse through poorly defined mechanisms. Myeloma cells inevitably develop chemoresistance that leads to disease relapse and patient-related deaths. Studies in tumor cell lines and biopsies obtained from patients refractory to therapy have revealed that myeloma cells adapt to stress by inducing expression of glucose-regulated protein 78 (GRP78), an endoplasmic reticulum (ER) chaperone with anti-apoptotic properties. Treatment of myeloma cells with bortezomib increased GRP78 levels and activated GRP78-dependent autophagy. Expression profiling indicated that GRP78-encoding *HSPA5* was significantly upregulated in bortezomib-resistant cells. Co-treatment with the anti-diabetic agent metformin suppressed GRP78 and enhanced the anti-proliferative effect of bortezomib. Bortezomib treatment led to GRP78 co-localization with proteotoxic protein aggregates, known as aggresomes. Pharmacologic suppression, genetic ablation or mutational inactivation of GRP78 followed by bortezomib treatment led to the accumulation of aggresomes but impaired autophagy and enhanced anti-myeloma effect of bortezomib. GRP78 was co-immunoprecipitated with the KDEL receptor, an ER quality control regulator that binds proteins bearing the KDEL motif to mediate their retrieval from the Golgi complex back to the ER. Taken together, we demonstrate that inhibition of GRP78 functional activity disrupts autophagy and enhances the anti-myeloma effect of bortezomib.

## INTRODUCTION

Multiple myeloma (MM) is the second most common cancer of the blood and, despite recent advances, remains incurable in the vast majority of patients [1, 2]. In the United States, there will be an estimated 24,050 new cases of MM in 2014 and >60,000 individuals living with the disease [3, 4]. Worldwide, ~86,000 patients are diagnosed each year with myeloma, while ~63,000 patients die every year from disease-related complications [5, 6]. Novel agents, such as the proteasome inhibitor bortezomib and the immunomodulatory agents thalidomide and lenalidomide, have extended overall survival (OS) to a median of ~7 years [7, 8]. Moreover, integration of novel agents into consolidation and maintenance therapy has further increased depth of response, including molecular complete responses, prolonged progression-free survival and OS. However, all MM patients eventually relapse and new molecular targets and more effective therapeutics represent an urgent, unmet need.

Drug resistance, either *de novo* or acquired, remains a significant obstacle in myeloma treatment [9, 10]. Our current knowledge of the genetic and epigenetic bases of therapeutic resistance remains poorly understood [11]. The ubiquitin (Ub)+proteasome system (UPS) is a complex protein network that maintains proteostasis through the selective degradation of misfolded, aggregated and short-lived proteins [12,13]. The proteasome serves as the catalytic core of the UPS to efficiently remove Ub-conjugated proteins and to maintain cell viability. The pivotal role of the proteasome in maintaining proteostasis has been exploited therapeutically to promote tumor cell death [14-16]. Bortezomib has emerged as the standard-of-care therapy for MM and catapulted the UPS into a position of prominence in cancer biology and drug development [14-18]. However, the mechanistic bases of resistance remains poorly understood.

Cancer cells adapt to proteasome inhibitors through induction of compensatory protein clearance mechanisms, e.g., aggresomes and autophagosomes, leading to the generation of drug resistance, therapeutic failure and disease relapse. Aggresomes are peri-nuclear structures formed in response to cellular stresses, such as hyperthermia, overexpression of insoluble or mutant protein and UPS inhibitors, that generate misfolded or partially denatured protein [19-21]. Histone deactylase (HDAC)6 and the microtubule-based motor protein, dynein, promote aggresome formation as a cytoprotective response that sequesters potentially cytotoxic protein aggregates. These structures then serve as a staging center for the delivery of protein aggregates to autophagosomes and eventual lysosomal removal.

ER stress induces autophagosome formation and has been shown to require components of the unfolded protein response (UPR) [22]. The glucose-regulated protein and molecular chaperone GRP78, is a major target upregulated during the UPR [22-24]. GRP78 is involved in translocating newly synthesized polypeptides across the ER membrane, facilitating their folding and assembly, maintaining proteins in a state competent for subsequent folding and oligomerization [25, 26]. GRP78 is also required for stress-induced autophagy [22-25]. Here, we reveal that GRP78 is required for the efficient delivery of bortezomib-induced aggresomes to autophagosomes and that targeting GRP78 holds promise as a strategy to overcome drug resistance in myeloma.

## RESULTS

### Expression of GRP78-encoding HSPA5 in MM patients and bortezomib-resistant cells

**Figure 1:**
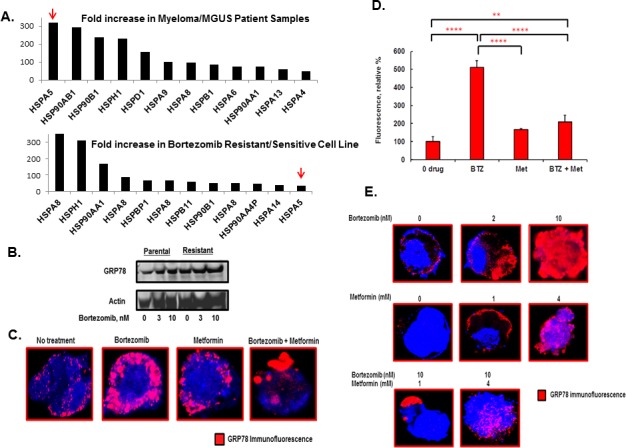
Effect of bortezomib and metformin treatment on GRP78 in myeloma cells A. Shown is the Fold-increase in the expression of individual HSP pathway genes in MM patient samples compared to MGUS samples (top panel). Also shown is the fold-increase in the expression of individual HSP pathway genes in bortezomib resistant RPMI8226 cells relative to drug-naïve cells (bottom panel). Shown is the fold-increase in relative expression determined by microarray-based profiling using Affymetrix 3.0 chips. B. Western blot comparing GRP78 levels in parental and bortezomib resistant cells. Parental and bortezomib resistant were grown in bortezomib for 18 hours prior to preparation of cell lysates. C. GRP78 staining of myeloma cells by IHC and confocal microscopy in RPMI8226 cells that had been treated with bortezomib (10nM), metformin (1mM) or both agents. Cells were treated for 18 hours under standard growth conditions. D. Quantitation of GRP78 levels based upon the relative level of fluorescent intensity detected by IHC staining. E. GRP78 staining RPMI8226 cells by IHC and confocal microscopy treated with various concentrations of bortezomib and metformin for 18 hours. Shown are representative images obtained from the same experiment performed multiple times.

The molecular chaperone GRP78 is induced under stress conditions such as glucose starvation, hypoxia and oxidative stress, which are characteristic of the tumor microenvironment. Levels of GRP78 are elevated in a variety of tumors, including prostate, lung, breast, colon and gastric tumors, myeloma and leukemias and GRP78 expression is inversely correlated with cancer patient survival [22, 23, 27]. A prior study analyzed bone marrow samples from 10 patients with Waldenström's macroglobulinemia (WM), 12 with MM and 11 with chronic lymphocytic leukemia (CLL) to show that *HSPA5* expression was increased relative to normal PCs obtained from healthy donors in these plasma cell disorders (GSE66910) [28]. We performed expression analysis to determine whether *HSPA5* expression was upregulated in patients with the pre-malignant condition monoclonal gammopathy of unknown significance (MGUS) that nearly uniformly precedes MM. Approximately 1-2% of MGUS patients per year will progress to develop MM and then require therapy. *HSPA5*, which encodes GRP78, was the HSP most significantly increased HSP-encoding gene detected in MM patient samples compared to MGUS samples (Figure 1A, top panel). Bortezomib resistant myeloma cells were then generated by treating RPMI8226 myeloma cells with either vehicle (0.5% DMSO) or successively increased concentrations of bortezomib. After six months of bortezomib exposure, drug exposed cells were 5-10 fold less sensitive to the proteasome inhibitor than the drug-naïve parental cells based upon IC_50_ values [18]. *HSPA5* expression was significantly upregulated in bortezomib-resistant cells (Figure 1A, bottom panel) and western blotting indicated that GRP78 was increased in bortezomib resistant cells compared to drug-naïve cells (Figure 1B). We reasoned that GRP78 upregulation could promote drug resistance through the induction of autophagy as a mechanism to eradicate potentially proteotoxic aggresomes. Activation of the UPR induces autophagy as a homeostatic mechanism triggered in response to misfolded protein accumulation in the ER lumen [29-31]. Bortezomib (10nM) treatment of myeloma cells led to a dramatic induction of GRP78 that accumulated near the cell membrane as shown by immunohistochemistry (IHC) and confocal microscopy (Figure 1C, D). Physiologic concentrations of metformin have been shown to suppress the UPR [32-35]. Since bortezomib induced GRP78 and metformin had been reported to target GRP78, we determined the metformin (1mM) effect on GRP78 levels combined with bortezomib (10nM) treatment. While bortezomib treatment alone significantly increased GRP78, metformin alone or co-treatment with bortezomib suppressed GRP78. The bortezomib effect on GRP78 was maximal at 10nM and the metformin effect was observed at 1 to 4mM (Figure 1E).

**Figure 2:**
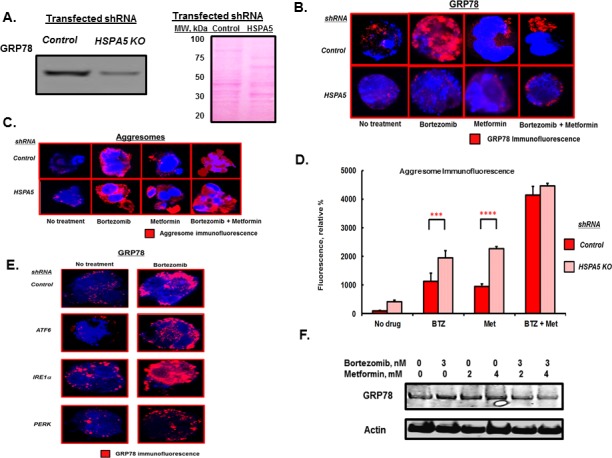
*HSPA5* knockdown effect on aggresome formation A. Western blot of GRP78 levels in lysates from RPMI8226 cells transfected with control or *HSPA5*-specific shRNA. Ponceau staining of the membrane used for the GRP78 blot is shown. B. RPMI8226 cells transfected with either scrambled control or *HSPA5*-specific shRNA were treated with drugs as indicated and the level of GRP78 determined by IHC and confocal microscopy. C. RPMI8226 cells transfected with either scrambled control or *HSPA5*-specific shRNA were treated with drugs as indicated for 18 hours and aggresomes visualized by IHC and confocal microscopy. D. Quantitation of aggresome levels based upon the relative level of fluorescent intensity detected by IHC and confocal microscopy. E. RPMI8226 cells transfected with scrambled control or shRNA to inactivate the stress transducers *ATF6, IRE1α* or *PERK* were treated with drugs as indicated and GRP78 levels determined by IHC and confocal microscopy. shRNA-mediated knockdown of the three stress transducers was validated by qRT-PCR. F. Western blot of myeloma cell lysate after treatment with bortezomib or metformin as indicted for 18 hours.

To investigate the role of GRP78 in aggresome formation, RPMI8226 cells were transfected with scrambled (control) shRNA or shRNA to inactivate *HSPA5*. Western blot indicated that shRNA to target *HSPA5* significantly reduced the level of GRP78 (Figure 2A). IHC and confocal microscopy indicated that bortezomib treatment significantly increased GRP78 in control-transfected cells but not in cells transfected with *HSPA5*-specific shRNA (Figure 2B). Metformin, alone or combined with bortezomib, suppressed the GRP78 induction (Figure 2B). However, bortezomib or metformin treatment of cells transfected with either control or *HSPA5*-specific shRNA led to a significant increase in aggresomes detected by using the proteostat dye-based method (*P*<*0.0001* for both Figure 2C, D). Aggresome formation in control cells treated with bortezomib was increased 11-fold compared to untreated cells *(P<0.001),* metformin treatment increased aggresomes by 8-fold compared to untreated cells *(P<0.004)* and bortezomib co-treatment with metformin increased aggresomes by 41-fold compared to untreated cells *(P<0.0001).* The results indicated that GRP78 upregulation was not essential for bortezomib-induced aggresome formation.

In addition to its chaperoning function, GRP78 is also a key regulator of the ER stress transducers inositol-requiring 1 (IRE1), activating transcription factor 6 (ATF6) and dsRNA-activated protein kinase-like ER kinase (PERK). GRP78 binds and inhibits IRE1, ATF6 and PERK activation in non-stressed cells [22-24]. Upon ER stress and misfolded protein accumulation in the ER, these molecules are released from GRP78 and become activated. The effect of bortezomib on GRP78 upregulation was observed in cells transfected with shRNA to inactivate either IRE1, ATF6 or PERK to suggest that the effect of bortezomib on GRP78 was independent of the three ER stress transducers (Figure 2E). Western blot confirmed that GRP78 levels were upregulated by bortezomib and that co-treatment with metformin suppressed the induction (Figure 2F).

**Figure 3:**
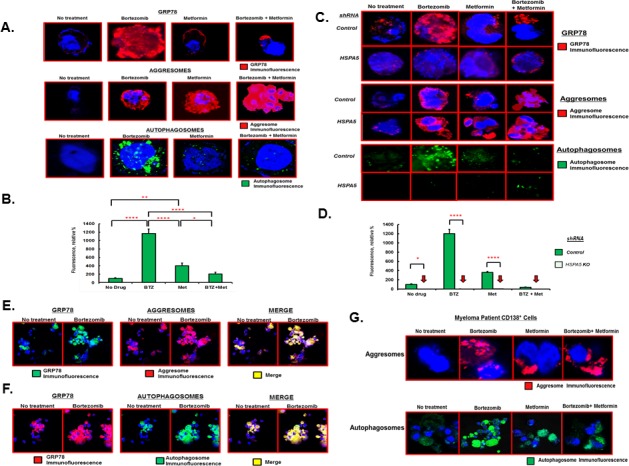
Effect of bortezomib and metformin on autophagosome formation A. RPMI8226 cells were treated with either bortezomib (10nM), metformin (1mM) or both agents for 18 hours under standard growth conditions. GRP78 was detected by IHC and confocal microscopy. Aggresomes and autophagosomes were detected using the dye-based methods. Shown are representative images seen on in at least three different experiments. B. Relative level of fluorescent intensity of autophagosomes after treatment of RPMI8226 cells with the indicated drugs. C. RPMI8226 cells were transfected with scrambled (control) or *HSPA5*-specific shRNA, treated with drugs as indicated and GRP78, aggresomes and autophagosomes detected as in Figure 3A. Shown are representative images seen on in at least three different experiments. D. Relative level of fluorescent intensity of autophagosomes after treatment of RPMI8226 cells transfected with either scrambled control or *HSPA5* shRNA and then treated with drugs as indicated. E. Co-localization of GRP78 with aggresomes as determined by IHC and confocal microscopy. RPMI8226 cells were treated with bortezomib (10nM), metformin (1mM) or both and stained using a GRP78-specirfic antibody, for aggresomes using dye-based reagent or both the GRP78 antibody and the dye-based reagent. Shown are representative images from multiple experiments. F. Co-localization of GRP78 with autophagosome as determined by IHC and confocal microscopy. RPMI8226 cells were treated with bortezomib (10nM), metformin (1mM) or both and stained using a GRP78-specirfic antibody, for autophagsomes using dye-based reagent or both the GRP78 antibody and the dye-based reagent. Shown are representative images from multiple experiments. G. Effect of bortezomib and metformin on aggresomes and autophagosomes in MM patient tumor cells. Patient bone marrow was obtained, CD138^+^ cells purified, treated with drugs as indicated and aggresomes and autophagosomes detected using the dye-based methods and confocal microscopy.

Bortezomib treatment of myeloma cells significantly increased not only GRP78 and aggresomes but also autophagosomes (Figure 3A). Treatment with metformin alone or co-treatment with bortezomib significantly increased aggresomes as well. However, metformin or co-treatment with bortezomib did not induce GRP78 or autophagosome levels. Bortezomib treatment increased green fluorescence ~12-fold compared to untreated cells (*P<0.0001*) while metformin treated cells exhibited a ~4-fold increase in green fluorescence compared to untreated cells (*P<0.001*) (Figure 3B). Bortezomib and metformin co-treatment led to fluorescence intensity less than that seen with bortezomib *(P<0.0001)* or metformin treatment alone *(P<0.02)*. The results suggested that metformin suppressed GRP78 upregulation and that GRP78 was required for bortezomib-induced autophagy.

To further investigate the role of GRP78 in autophagy, *HSPA5* was genetically inactivated in RPMI8226 cells by shRNA-mediated knockdown and drug effects were determined alone or in combination (Figure 3C). Bortezomib increased GRP78 in cells transfected with control shRNA but not in cells transfected with *HSPA5* shRNA. Bortezomib treatment alone, or combined with metformin, promoted aggresome formation in both control cells and cells with *HSPA5* inactivated. A punctate pattern of green fluorescent cytoplasmic structures, characteristic of autophagosomes, was seen and represented the accumulated autophagosomes. Similar to the metformin effect, genetic inactivation of *HSPA5* impaired bortezomib-induced autophagosome formation (Figure 3C, D). Bortezomib increased green fluorescence in control transfected cells by ~12-fold compared to untreated cells. Bortezomib treatment of cells transfected with *HSPA5* shRNA did not increase green fluorescence (Figure 3D). GRP78 co-localized with both aggresomes and autophagosomes (Figure 3E, F). The effects of bortezomib and metformin on aggresome and autophagosome formation were also observed using myeloma patient CD138^+^ cells that had been purified from patient bone marrow biopsies (Figure 3G).

**Figure 4:**
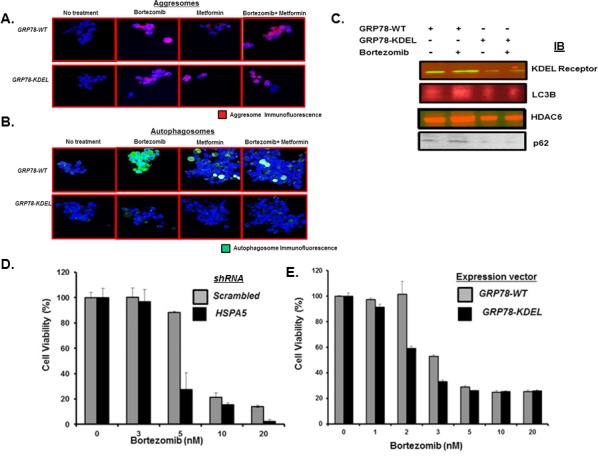
Effect of GRP78 genetic silencing on bortezomib-induced autophagosome formation A. U266 cells were transfected with plasmids that expressed either control (pcDNA3.1) or a GRP78 mutant (P495L). Cells were treated with bortezomib (10nM), metformin (1mM) or both for 18h. Aggresomes were detected by the by dye-based method. Shown are representative images from multiple experiments. B. U266 cells were transfected with plasmids that expressed either control (pcDNA3.1) or the GRP78 mutant. Cells were treated with bortezomib (10nM), metformin (1mM) or both for 18h. Autophagosomes were detected by dye-based methods. Shown are representative images. C. U266 cells were transfected with plasmids that expressed GRP78-WT or the GRP78 mutant. Cells were treated with bortezomib, lysates immunoprecipitated and probed by western blot to detect the association of aggresome (p62 and HDAC6) or autophagosome pathway (KDEL receptor and LC3B) effectors with GRP78. D. U266 cells were transfected with plasmids that expressed either shRNA to inactivate control (scrambled) or *HSPA5.* Cells were treated with bortezomib at indicated concentrations and the effect on viability determined using the XTT assay. Values represent the mean of triplicate measurements and error bars represent the standard deviation (SD). E. U266 cells were transfected with plasmids that expressed either GRP78-WT or the GRP78 mutant. Cells were treated with bortezomib as indicated and the effect on viability determined using the XTT assay. Values represent the mean of triplicate measurements and error bars represent the SD.

We reasoned that bortezomib promoted formation of aggresomes that were delivered in a GRP78-dependent manner to autophagosomes. Myeloma cells were transfected with plasmids that expressed either wildtype (WT) GRP78 or a defective version with the substrate binding domain mutated. The KDEL receptor is an ER quality control protein that binds chaperone proteins containing the KDEL motif, e.g., GRP78, calnexin and protein disulfide isomerase, to mediate their ER retrieval from post-ER compartments, e.g., the Golgi complex, back to the ER. Cells transfected with plasmids expressing GRP78-WT or the GRP78 mutant readily formed aggresomes after bortezomib treatment as well as after metformin or bortezomib and metformin co-treatment (Figure 4A). However, after bortezomib treatment, cells transfected with the GRP78 mutant did not form autophagosomes as readily as did cells transfected with GRP78-WT (Figure 4B). To identify aggresome and autophagosome proteins that interacted with GRP78, myeloma cells transfected with c-myc-tagged GRP78-WT or GRP78 mutant were treated with bortezomib, lysates prepared, immunoprecipitated with a myc-specific antibody and probed by western blot. Blots indicated that GRP78-WT, but not the GRP78 mutant, associated with the KDEL receptor and LC3B as well as the aggresome components HDAC6 and p62 (Figure 4C). Cells transfected with shRNA to knockdown *HSPA5* were more sensitive to bortezomib than controls and cells that expressed the GRP78 mutant were more sensitive to bortezomib than control-transfected cells (Figure 4D, E).

**Figure 5:**
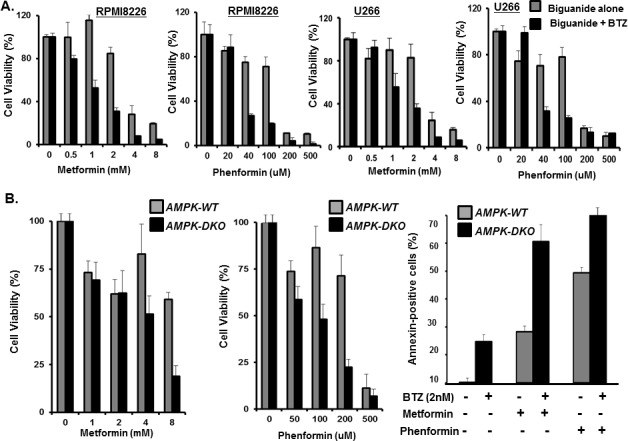
Metformin effect on myeloma viability A. Dose dependent effect of the biguanides alone or combined with bortezomib on myeloma proliferation. RPMI8226 and U266 cells were incubated with the biguanides alone or biguanides and bortezomib (2nM) for 72 hours. Bortezomib alone at 2nM yielded ~10% reduction in cell viability. Proliferation was determined using the XTT assay and error bars represent SD values determined from triplicate measurements. B. Effect of biguanide in *AMPK-WT* and *AMPK-DKO* MEF proliferation measured using the XTT assay and error bars represent SD values determined from triplicate measurements. Annexin-positive cells were quantitated by flow cytometry.

Metformin is a widely used antidiabetic agent that decreases insulin resistance and lowers blood glucose levels through inhibition of liver glucose production and an increase in glucose uptake in muscles [36-39]. Metformin has also been shown to reduce the viability of numerous cancer cell lines and to inhibit the progression and relapse of breast, prostate and lung cancer mouse xenografts, when combined with suboptimal doses of standard chemotherapeutic agents [40-43]. Diabetic patients treated with metformin have a reduced incidence of cancer and cancer-related mortality [44-46]. Since metformin suppressed stress-induced elevation of GRP78, we determined the metformin effect on the anti-proliferative effect of bortezomib. Metformin or phenformin treatment reduced myeloma proliferation and co-treatment with bortezomib synergistically increased the anti-proliferative effect of the proteasome inhibitor (Figure 5A). The anti-proliferative effects of metformin and phenformin were also observed in mouse embryonic fibroblasts (MEFs) that expressed either AMPK WT or AMPK DKO (Figure 5B). Functional inactivation of AMPK did not eliminate the metformin effect on myeloma cells to suggest that the effects were mediated, at least in part, through AMPK-independent processes. Our results are consistent with a number of prior studies that indicate that metformin induces cellular stress and apoptosis through AMPK-independent pathways [47-49]. However, we cannot exclude that low, residual AMPK activity is sufficient to mediate the effect of metformin under certain conditions or that alternate pathways are activated upon metabolic stress in cells that lack functional AMPK. The inhibitory effect on myeloma proliferation appears to be not only AMPK-independent, but must be independent of metformin's insulin-sensitizing and anti-hyperglycemic effects since they were observed in cell-based assays.

**Figure 6:**
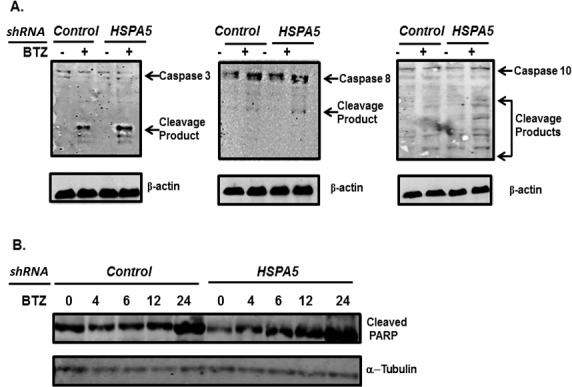
Effect of *HSPA5* knockdown on bortezomib-induced cleavage of caspases and PARP A. U266 cells were transfected with plasmids that expressed either shRNA to control (scrambled) or *HSPA5.* Cells were treated with bortezomib, lysates prepared and probed by western blot using antibodies to Caspase 3, 8 and 10. B. U266 cells were transfected with plasmids that expressed either shRNA to control (scrambled) or *HSPA5.* Cells were treated with bortezomib, lysates prepared and probed by western blot using antibodies to PARP.

Bortezomib triggered apoptosis is mediated through activation of poly (ADP) ribosome polymerase, caspase-3 and caspase-8 [50]. Myeloma cells were transfected with shRNA to knockdown *HSPA5*, treated with bortezomib and the effect on cleavage of PARP and caspase determined by western blot (Figure 6A, B). PARP and caspases-3 and -8 were more readily cleaved in cells that lacked *HSPA5* compared to control-transfected cells. In addition, caspase-10 has been reported to modulate the autophagic response. Consequently, preventing caspase-10 cleavage promotes myeloma cell survival [51]. Following bortezomib treatment, caspase-10 was also more readily cleaved in cells that lacked *HSPA5* than in control-transfected cells.

## DISCUSSION

The ER molecular chaperone GRP78 associates with and promotes the delivery of bortezomib-induced aggresomes to autophagosomes to promote their efficient disposal. Pharmacologic or genetic suppression of GRP78 followed by proteasome inhibition significantly impaired autophagosome formation. The ER is a putative membrane source for generation of autophagic vacuole membranes and is massively expanded and disorganized in cells where GRP78 is suppressed [52]. Upon ER stress, unfolded proteins accumulate in the ER and are cleared by the proteasome-involved ER-associated degradation system. We hypothesized that GRP78-mediated autophagy served as an alternative protein clearance mechanism that circumvented inhibition of the UPS to promote drug resistance in tumor cells. The findings provide a new mechanism for the protective function of GRP78 in stressed cells to promote drug resistance to suggest that GRP78 is required for bortezomib-induced autophagy. GRP78 represents an actionable, “druggable”, anti-cancer target to overcome drug resistance.

The orally-administered, well-tolerated and inexpensive anti-diabetic agent metformin displayed anti-myeloma effects and enhanced the anti-proliferative of bortezomib. Metformin is first-line therapy used worldwide by millions of type 2 diabetes (T2D) patients. Epidemiological studies have reported that a subset of T2D patients treated with metformin had a lower cancer risk and reduced cancer-related mortality rates compared to those treated with other agents [53,54]. Furthermore, metformin has demonstrable antitumor activity in xenografts, carcinogen-induced and genetically-engineered mouse models to support pharmacologic repositioning for anti-cancer therapy. The results suggest that bortezomib co-treatment with metformin offers a therapeutic advantage for myeloma patients over the use of either agent alone.

Consistent with prior studies in other model systems, we found that the anti-tumor effect of phenformin was significantly greater than that of metformin. The IC_50_ values for metformin determined here were in the millimolar range, much greater than that observed for phenformin and much higher than the maximally-achievable level following oral administration in humans [55]. However, the risk/benefit ratio clearly favors metformin over phenformin for treatment of T2D. Historically, the daily administration of phenformin was associated with an elevated incidence of lactic acidosis and was linked to fatal outcomes leading to removal from US markets. Currently there are >60 clinical studies evaluating the anticancer effects of metformin or the preventive effects in patients at high cancer risk. Future studies will clinically evaluate bortezomib combined with metformin in myeloma patients.

We hypothesized that pharmacologic inhibition of autophagy could be achieved using metabolic stress-inducing agents to enhance the anti-myeloma effect of bortezomib. Functional inactivation of AMPK genetic ablation did not eliminate the effect of metformin to suggest that the metformin effects are mediated, at least in part, through AMPK-independent processes. Our results are consistent with prior studies to indicate that metformin induces cellular stress and apoptosis through AMPK-independent pathways. However, we cannot exclude the possibility that low, residual AMPK activity is sufficient to mediate the effects of metformin under certain conditions or that alternate pathways are activated upon metabolic stress in cells that lack functional AMPK. The inhibitory effect of metformin on myeloma proliferation appears to be not only AMPK-independent, but must be independent of metformin's insulin-sensitizing and anti-hyperglycemic effects since these effects were observed in cell-based assays.

The findings reported here should be interpreted in the context of the available data about the protective effect of GRP78 on cancer cells to promote a chemoresistant phenotype and metastasis that has been associated with poor clinical outcomes [56-58]. Specifically, GRP78 is overexpressed in response to ER-stress induced cancer treatments and intracellular GRP78 accounts for 90% of the total cellular content and its upregulation causes re-localization of GRP78 to cell membrane [59]. Transmembrane GRP78 acts through the PI3K/AKT pathway to promote cell survival and metastasis and the humanized antibody against GRP78, MAb159, is promising to enter in early therapeutic trials [60]. However, our study suggests that pharmacologic suppression of GRP78 may also suppress GRP78 induction and transmembrane localization. The crucial role of energy metabolism in cell growth and proliferation implies that antidiabetic or metabolism-altering drugs may hold preventive and therapeutic value in cancer54. Because of its exceptionally low toxicity profile, FDA approval and early signs of efficacy, metformin is currently at the forefront of this drug class. Clinical trials with metformin in non-diabetic patients are needed to illuminate the potential use of this drug in MM. Pharmacologic repositioning of FDA-approved agents may reduce the risk of failure in the drug discovery process as well as the costs associated with *de novo* drug development [61]. In summary, the therapeutic promise of metabolic enhancers in the non-diabetic setting or other emerging forms of therapy that target protein degradation pathways may enhance the cytotoxic efficacy of bortezomib [62, 63].

## MATERIALS AND METHODS

### Cell lines and reagents

MM cell lines (MMCLs) were from the National Cancer Institute, Bethesda, MD and cultured as described^16-18^. BM aspirates were obtained from patients after approval by the UC Cancer Institute Institutional Review Board. Malignant PCs were purified by positive CD138 microbead selection (Miltenyi Biotec, San Diego, CA). Bortezomib was from ActiveBiochem (Maplewood, NJ), metformin (1,1–dimethyl biguanide hydrochloride), phenformin: N-(2-Phenylethyl) imidodicarbonimidic diamide monohydrochloride, phenethyl-biguanide) and reagent grade chemicals from Sigma Chemical Co. (St. Louis, MO). Plasmids pCMV-GRP78-myc-WT or pCMV-GRP78-myc-P495L were from Addgene (Cambridge, MA).

### Generation of bortezomib-resistant cells

RPMI8226 cells were exposed to successively increased concentrations of bortezomib to generate drug resistant cells. Parental cells were cultured under the same algorithm in vehicle (0.5% DMSO) alone.

### Gene Expression Microarray

Total RNA was isolated from MMCLs using the miRNeasy kit (Qiagen, Inc., Germantown, MD). Samples were hybridized to the Genechip primeview human gene expression array and those demonstrating a cutoff greater or less than 2-fold difference from normal PC were used for further analysis. RNA quality was confirmed using the Agilent 2100 Bioanalyzer. For each sample, the 3′ *in vitro* translation express kit (Affymetrix, Santa Clara, CA) synthesized biotin-labeled RNA target from 100ng of total RNA.

### Gene knockdown

shRNA in the pLKO.1-TCR cloning vector were from the Lenti-shRNA Library Core (Cincinnati Children's Hospital Medical Center, Cincinnati, OH). Lentiviral vectors were transfected into 293T cells with packaging (4ug) and envelope vectors (4ug) using Lipofectamine-2000 (Invitrogen). Virus was concentrated by ultra-centrifugation and resuspended in PBS at ~1×10^9^ infectious units/mL to transduce myeloma cells then grown under puromycin (0.5ug/mL) selection for >2 weeks.

### Western blotting

Cells were pelleted, resuspended in RIPA buffer (Sigma) containing protease inhibitor cocktail and phosphatase inhibitors (Cell Signaling Technology, Danvers, MA), centrifuged, protein concentration determined, sample buffer added, boiled and loaded onto gels (Wako Chemical, Richmond, VA). Proteins were transferred to nitrocellulose and incubated with primary antibody (1:1000) overnight at 4^o^C. Bands were visualized using LI-COR (Littleton, CO) IRDye anti-mouse IgG or anti-rabbit secondary antibodies in the Odyssey detection system. Antibodies to GRP78, HDAC6 and KDEL receptor were from Abcam, LC3B and p62 from Cell Signaling Technology and Alexa Fluor647 from Invitrogen.

### GRP78 detection

Myeloma cells were treated under standard growth conditions, pelleted, washed with PBS containing 0.1% BSA, fixed with 4% paraformaldehyde and permeabilized by PBS containing 0.5% Triton X-100 (PBST), 3 mM EDTA, pH 8. Cells were washed twice, blocked with 1% BSA in PBST for 30min at room temperature and incubated with a GRP78-specific rabbit polyclonal antibody (Abcam) for 1 hour at room temperature. Cells were washed, incubated with anti-rabbit Alexa fluor647 (Invitrogen), washed and applied with DAPI fluoromount-G (Southern Biotech Birmingham, AL) to slides and visualized using a Zeiss LSM170 confocal microscope with settings for DAPI (EX 405nm, EM 410-480nm) and red fluorescent protein (EX 633nm, EM 650-700nm).

### Aggresome detection

The proteostat aggresome detection kit (Enzo Life Science, Farmingdale, NY) was used to visualize aggresomes by confocal microscopy. MM cells were plated, treated with bortezomib and metformin at the indicated concentrations under standard conditions, collected, centrifuged, washed, supernatant discarded and the pellet fixed, washed, permeabilized, washed again then incubated in 100μL of dual detection reagent for 30 minutes in the dark. Excess detection reagent was removed and the sample suspended in 100μl assay buffer, applied to a microscope slide and analyzed using a Zeiss LSM170 confocal microscope with the settings for DAPI (EX 405nm, EM 410-480nm) and Texas Red (EX 560nm, EM 600-650)

### Autophagosome detection

The cyto-ID autophagy detection kit (Enzo Life Science) was used to visualize autophagosomes by confocal microscopy. MM cells were plated, treated with bortezomib and metformin at the indicated concentrations under standard conditions, collected, reagent and Hoechst stain for 30 minutes in the dark. Excess detection reagent was removed and the sample suspended in 100μl assay buffer. The cell suspension was applied to a glass microscope slide and analyzed using a Zeiss LSM170 confocal microscope with the settings for DAPI (EX 405nm, EM 410-480nm) and FITC (EX 488nm, EM 500-550nm)

### Quantitation of fluorescence

Fluorescent IHC images were collected by using a Zeiss LSM710 Confocal Laser Scanning Microscope with a 63x/1.2 objective lens. Fluorescence was quantified using ImageJ (NIH, Bethesda, MD).

### Cell viability

MM and patient tumor cell viability was measured using the XTT (Sigma) dye absorbance. 5×10^3^ cells were plated in 96-wells and incubated in media that lacked phenol red, treated with drugs and incubated for 72 hours. XTT-PMS mixture was added, plates incubated for 4 hours and absorbance determined using a BMG Labtech Fluostar Optima plate reader.

### Quantitation of apoptosis

1×10^6^ cells were cultured for 24 hours with bortezomib and either biguanide. Cells were harvested, washed, and stained with annexin V/propidium iodide as described. Annexin V^+^/PI^−^ apoptotic cells were enumerated using the Epics flow cytometer. The percentage of cells undergoing apoptosis was defined as the sum of early apoptosis (annexin V^+^) and late apoptosis (annexin V^+^ and PI^+^) cells.

### Statistical analysis

All *in vitro* experiments were performed in triplicate, repeated at least twice and a representative experiment was selected for presentation. The statistical significance of differences was determined using the ANOVA test with a *P* <0.05. All statistical analyses were determined using Graph Pad Prism 6 Software (San Diego, CA).
